# Self-assessed life expectancy among older adults in Côte d’Ivoire

**DOI:** 10.1186/s12889-020-09034-4

**Published:** 2020-06-15

**Authors:** Richard K. Moussa, Vakaramoko Diaby

**Affiliations:** 1 Théorie économique, modélisation et applications (ThEMA), Université de Cergy-Pontoise, France and Ecole Nationale Supérieure de Statistiques et d’Economie Appliquée (ENSEA), 08 BP 03 Abidjan 08, Abidjan, Côte d’Ivoire; 2 Pharmaceutical Outcomes & Policy (POP), College of Pharmacy, University of Florida, HPNP 3317, 1225 Center Drive, Gainesville, FL 32610 USA

**Keywords:** Subjective life expectancy, Self-assessed survival probabilities, Finite mixture models, Elderly, Côte d’Ivoire

## Abstract

**Background:**

The purpose of this study was to estimate individuals’ expected longevity based on self-assessed survival probabilities and determine the predictors of such subjective life expectancy in a sample of elderly people (50 years and older) in Côte d’Ivoire.

**Methods:**

Paper-based questionnaires were administered to a sample (*n* = 267) of older adults residing in the city of Dabou, Côte d’Ivoire in May 2017. Information on subjective expectations regarding health, comorbidities, and self-assessed survival probabilities was collected. We estimated self-assessed life expectancy and its determinants using a two-pronged approach by: (i) estimating individuals’ life expectancy using the self-assessed survival probabilities (SSPs), and (ii) applying a finite mixture of regression models to form homogenous groups of individuals (clusters/components) and investigate the determinants.

A spline-based approach was used to estimate the overall distribution of life expectancy for each individual using two to four points of self-assessed survival probabilities. A finite mixture of regression models was used to identify homogeneous groups of individuals (i.e. clusters/components) of the overall subjective life expectancy distribution of the study participants.

**Results:**

The mean subjective life expectancy in older people varied according to four components/clusters. The average subjective life expectancy among the elderly was 79.51, 78.89, 80.02, and 77.79 years in the first, second, third, and fourth component of the subjects’ overall subjective life expectancy, respectively. The effect of sociodemographic characteristics, comorbidities, and lifestyle on subjective life expectancy varied across components. For instance, a U-shape relationship between household per capita income and subjective life expectancy was found for individuals classified into the third component, and an inverse U-shape relationship was found for individuals classified into the fourth component.

**Conclusions:**

We extended the estimation of subjective life expectancy by accounting for heterogeneity in the distribution of the estimated subjective life expectancy. This approach improved the usual methods for estimating individual subjective life expectancies and may provide insight into the elderly’s perception of aging, which could be used to forecast the demand for health services and long-term care needs.

## Background

Studies using subjective life expectancy (SLE) as a measure of longevity have stressed that SLE is an important determinant of changes in labor supply, health, and consumption behaviors [1–3]. SLE determines the intended retirement age [1] and the way older workers prepare for their retirement [2]. There is evidence that older workers take into consideration SLE in their decision to postpone retirement [2]. Even among retirees, SLE affects the decision to return to paid work [2]. Furthermore, SLE was shown to affect health self-regulation [3]. Individuals with low SLE have a weak intention to perform physical exercises, and when they do, they are less likely to plan for those exercises. SLE was also been shown to affect bequest, consumption, and savings [4]. Although important, given its social and economic implications, research on SLE in developing nations is lacking, particularly for the elderly.

This paper focuses on the case of a sub-Saharan African country, Côte d’Ivoire. Côte d’Ivoire is a developing country with an average life expectancy of 56.8 years (Population and Housing Census of 2014) [5]. The poverty rate is relatively high (46.3% in 2015), with 30% of the overall population living in slums [6]. Several non-communicable and communicable illnesses that include Human Immunodeficiency Virus/Acquired Immune Deficiency Syndrome (HIV/AIDS), tuberculosis, blood pressure, are highly prevalent, and unhealthy lifestyle such as smoking or alcohol consumption are also developing [7]. All these factors tend to affect individuals’ health conditions, and thus, their SLE. An individual’s SLE is key for understanding individual consumption choices and behaviors, especially for older individuals [8, 9], and can potentially lead to the development of specific policies to address aging. However, measuring SLE is not a straightforward task.

Several studies use the self-assessed survival probabilities (SSPs), i.e. assessment of the probability of surviving to a certain predefined age [4], to determine the time until the end of life. Previous studies established that the SSP is an independent predictor of real-life expectancy and mortality [8–11]. Besides, studies showed that the variance of the SSP declines with age [12]. Thus, it has been recommended that studies examining the end of life behaviors should use SLE instead of actuarial tables [6]. However, critics argue that the traditional approach for measuring the SLE using only one SSP fails to fully capture the expectations formation process of individuals who are subject to that exercise.

In this paper, we aim to (i) estimate the SLE of individuals aged 50 years and older using the two to four SSPs, and (ii) identify the determinants of such SLE in Dabou, a county in Côte d’Ivoire.

## Methods

### Data

Data were abstracted from the health section of a survey developed by students and researchers at the Ecole Nationale Supérieure de Statistique et d’Economie Appliquée (ENSEA - national school of statistics and applied economics in English) and administered to a sample of 15 to 65 years old in Dabou and its vicinity, a small town at 60 km from Abidjan, Côte d’Ivoire, in May 2017.

A multistage sampling strategy was used to collect data on *the living condition, resilience, and health among the elderly* in Dabou and its vicinity. We randomly selected households stratified according to the following categories: 1) group 1: Dabou (the town itself), 2) group 2: 4 villages (Dabou’s vicinity) with more than 700 households, 3) group 3: 3 villages with 400 to 700 households, 4) group 4: 8 villages with 150 to 400 households, and 5) group 5: 1 village with less than 150 households. We used the following respective sampling rate: 5, 20, 33, 50 and 100%. This drawing allowed us to obtain a sample of 2776 households, among which 663 from group 1 (Dabou), 794 from group 2, 427 from group 3, 823 from group 4, and 69 in from group 5. Then, at the level of each household, a simple random draw was used to capture one individual among those 15 and above in age.

Our sample size (*n* = 2775) was calculated to ensure that with an alpha of 0.05 and a power of 0.9, a rate of resilience of 50%, the minimum detectable variation is under 3%. However, due to a 9% nonresponse rate, the final sample size was *n* = 2523 for the entire survey. We extracted information from this dataset regarding individuals aged 50 to 65 (*n* = 267).

Two pre-tested questionnaires were used in the survey. The first questionnaire was administered to the head of the household to gather information on the members of the household, including demographic characteristics (e.g. gender, age), education and employment status, and dwelling/living conditions (Additional file 1). The second questionnaire (Additional file 2) gathered information about the participants’ health, including overall self-assessed health, conditions, physical functioning, health behaviors (e.g. smoking, alcohol, diet, physical exercise), and self-assessed survival probabilities for four target ages (70, 75, 80 and 85). Informed consent was obtained from all subjects (parent and/or legal guardian for participants under 18) before the administration of the survey. From the dataset created following the administration of questionnaires, we extracted information about all individuals aged 50 to 65 to target elderly populations.

### Measures

#### Outcome variable

SLE is the outcome of this study. This variable is not directly collected in the questionnaire of the survey but was estimated based on SSPs. The SSPs were assessed by four items:

- On a scale of 0 to 100, what do you estimate your chances of being alive at target age 70 is?
- On a scale of 0 to 100, what do you estimate your chances of being alive at target age 75 is?
- On a scale of 0 to 100, what do you estimate your chances of being alive at target age 80 is?
- On a scale of 0 to 100, what do you estimate your chances of being alive at target age 85 is?

#### Independent variables

We included a comprehensive list of independent variables informed by the literature [13, 14]. These variables were categorized into seven groups of variables: sociodemographic, economic, comorbidities, behavioral, quality of life, living conditions, and genetic information.

Sociodemographic variables: Age was a continuous variable, measured in years. Two gender categories were created (1 if male, 0 if female). Two living with partner categories were created (1 if lives with a partner, 0 otherwise). Two educational levels categories were created (1 if the respondent attended primary school at most, 0 otherwise).

Economic variables: Two employment categories were created (1 if the individual is employed, 0 otherwise). Income per capita was a continuous variable, measured in dollars.

Comorbidities: Two comorbidities variables were considered: (1) Obese [1 if obese (Body Mass Index ≥30 Kg/m2), 0 otherwise]; (2) Chronic condition (1 if individual reports at least one chronic condition, 0 otherwise).

Behavioral variables: Four categorical variables were used: (1) Current smoker (1 if the individual is currently a smoker, 0 otherwise), (2) Alcohol intake (1 if the individual consumes at least 2 glasses of alcohol per day, 0 otherwise), (3) Physical activity (1 if individual exercises, 0 otherwise), (4) Diet (1 if the individual observes a diet, 0 otherwise).

Quality of life variables: Three categorical variables were created: (1) health conditions limiting adults’ ability to perform activities of daily living (1 if the individual reports that his health condition limits his/her working ability, 0 otherwise), (2) Health status 1 (1 if individual reports poor health status, 0 otherwise), (3) Health status 2 (1 if the individual reports that his health is deteriorating compared to the previous year, 0 otherwise).

Living conditions variables: Three categorical variables were used: (1) Toilet with flush (1 if the individual has access to a flush toilet, 0 otherwise), (2) Underground water (1 if the individual uses underground water as drinking water, 0 otherwise), (3) Septic tank (1 if the individual uses a septic tank for wastes, 0 otherwise).

Genetic information variables: Three categorical variables were created: (1) Mother alive (1 if the individual’s mother is alive, 0 otherwise), (2) Father alive (1 if the individual’s father is alive, 0 otherwise), (3) Both parents alive (1 if the individual’s both parents are alive, 0 otherwise).

### Estimation of self-assessed life expectancy

We estimated SLE and its determinants using a two-pronged approach by (i) estimating individuals’ life expectancy using the self-assessed survival probabilities (SSPs) (approach by Bellemare et al. (2012)) [15], and (ii) applying a finite mixture of regression models to form homogenous groups of individuals (clusters/components) and investigate the determinants. A technical note on the methodological approach is presented in the Additional file 3.

The approach by Bellemare et al. is based on a cubic spline smoothing around each SSP value. This smoothing helps to estimate the cumulative distribution function of the SLE for each individual. Given that the cumulative distribution function is strictly monotonic, the function can be approximated by a cubic polynomial around each SSP. Subsequently, the cumulative distribution function of the SLE is estimated by connecting these local polynomials. Finally, the average life expectancy for each individual is calculated from the estimated cumulative distribution function. Suppose that a person reports 100 as its SSP for the target age of 70, 60 as its SSP for the target age of 75, 30 as its SSP for the target age of 80, and 0 as its SSP for the target age of 85. Then, on each of the intervals ([70; 75], [75; 80] and [80; 85]) the cumulative distribution function is approximated by a cubic polynomial and the junction of these polynomials provides the overall cumulative distribution function for the individual.

### Statistical analyses

For comparison purposes, we plotted the distribution of the estimated SLE for both males and females (stratified by age groups) using kernel density estimation. In the second step of our methodological approach, we used a finite mixture model to assess the determinants of the estimated life expectancy, accounting for the heterogeneity in the distribution of the life expectancy among elders. This heterogeneity exists because the perception of life expectancy can differ among elders, even if they have the same characteristics. The finite mixture model allows creating homogenous clusters of SLE for elders based on individual characteristics, which are called concomitant variables. Variables, such as self-assessed health status, self-assessed health variation, or having a parent alive might affect the individual’s SLE and would be used as concomitant variables. Then, instead of a constant effect of the determinants on SLE for the whole population, this framework allows the determinants of the SLE to vary across clusters.

The study was approved by the National Ethical Committee of Côte d’Ivoire (Comité Consultatif National de Bioéthique de la République de Côte d’Ivoire in French) and written informed consent forms were obtained from participants. All methods were carried out in accordance with relevant guidelines and regulations.

## Results

### Descriptive statistics

The dataset used consists of 267 individuals with an average age of 56.4 and predominantly males (55.4%). 60% of the individuals had a low education level. Table 1 provides additional statistics on the dataset. From these average values in Table 1, we observed that the obesity rate grows slightly with age (12% on average for individuals aged 50–54, 13% for individuals aged 55–59 and 19% for individuals aged 60–64). Moreover, alcohol consumption declined slightly with age, while the smoking rate only declined after the age of 60. Besides, the employment rate decreased with age (from 71% for those aged 50–54 to 36% for those aged 60–64). It is also notable that only a relatively small proportion of older people perform physical activity (only 21% of those aged 50–65 perform physical activity). Furthermore, for each age group, the SSP declined with the target age. However, for the same target age, the average SSP increased with age groups. At the target age of 75, for example, the average SSP was 60% for individuals aged 50–54, 66% for those aged 55–59, and 71% for those aged 60–64.

**Table 1:** Descriptive statistics

Variable	Aged 50–55 (SD)	Aged 55–60 (SD)	Aged 60–65 (SD)	All ages (SD)
Proportion (in %)	37.08	35.21	27.72	100
Age	51.94 (1.44)	56.79 (1.4)	61.92 (1.57)	56.41 (4.25)
Male	0.55 (0.5)	0.54 (0.5)	0.58 (0.5)	0.55 (0.5)
Obese	0.12 (0.33)	0.13 (0.34)	0.19 (0.39)	0.14 (0.35)
Rural	0.86 (0.35)	0.85 (0.36)	0.8 (0.4)	0.84 (0.37)
Low education level	0.60 (0.49)	0.59 (0.50)	0.61 (0.49)	0.60 (0.49)
Employed	0.71 (0.46)	0.6 (0.49)	0.36 (0.48)	0.57 (0.5)
Current smoker	0.08 (0.27)	0.1 (0.3)	0.05 (0.23)	0.08 (0.27)
Alcohol consumption	0.48 (0.5)	0.44 (0.5)	0.32 (0.47)	0.42 (0.49)
Physical activity (Exercise)	0.2 (0.4)	0.23 (0.43)	0.2 (0.4)	0.21 (0.41)
Time horizon (to 70)^a^	18.06 (1.44)	13.21 (1.4)	8.08 (1.57)	13.59 (4.25)
SSPs at target age 70	0.71(0.26)	0.76 (0.22)	0.76 (0.26)	0.74 (0.25)
SSPs at target age 75	0.6 (0.27)	0.66 (0.25)	0.71 (0.29)	0.65 (0.27)
SSPs at target age 80	0.53 (0.27)	0.58 (0.27)	0.63 (0.29)	0.57 (0.28)
SSPs at target age 85	0.45 (0.27)	0.48 (0.27)	0.56 (0.3)	0.49 (0.28)

*SD* Standard deviations

^a^Age 70 minus current age

### Estimation of self-assessed life expectancy

**Fig. 1:**
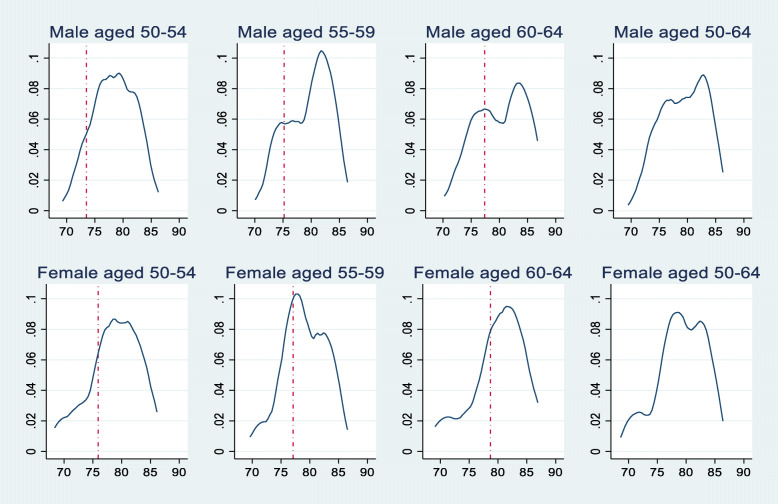
Estimated life expectancy per gender and age groups. Notes: The y-axis represents the density (probabilities). The blue curve is the kernel density of the estimated subjective life expectancy for the whole population (both males and females for all age groups) while the red line is the national level life expectancy

**Fig. 2:**
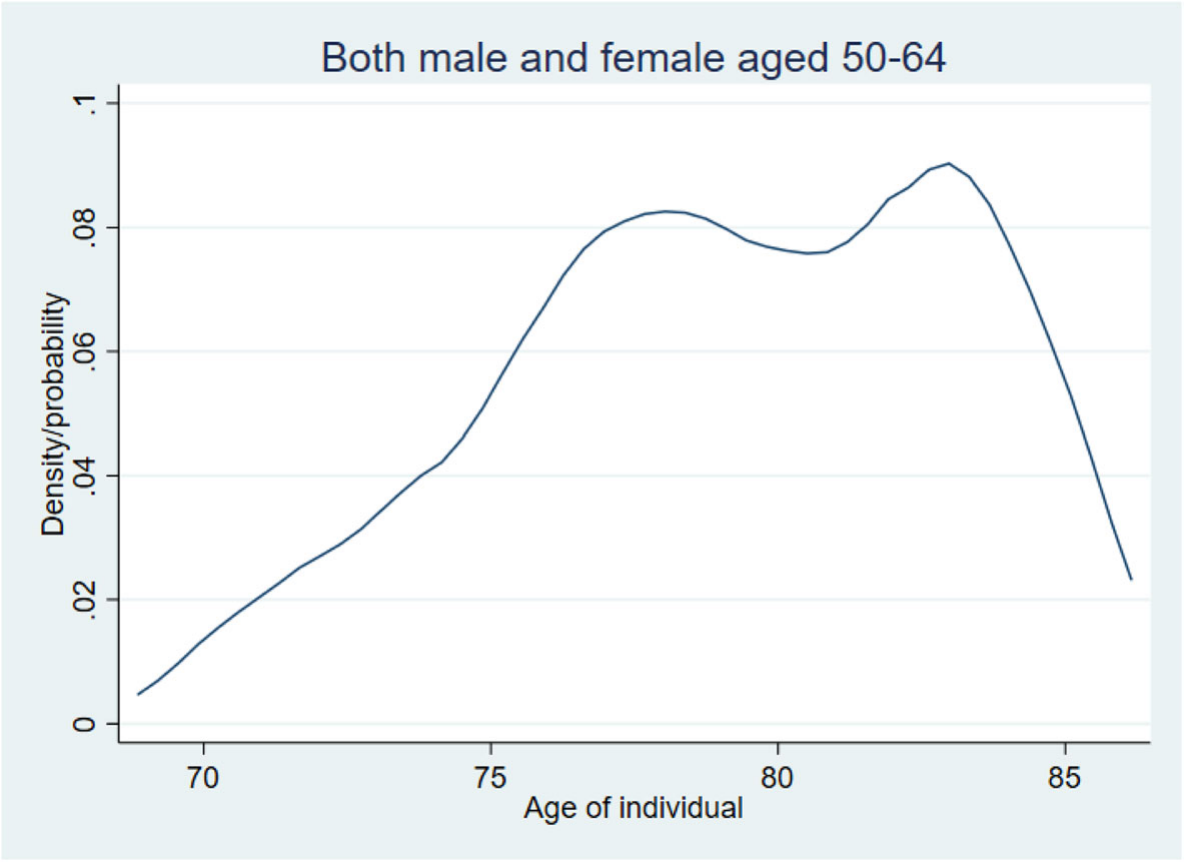
Estimated life expectancy of both males and females aged 50 to 64 years old. Note: The blue curve is the kernel density of the estimated subjective life expectancy for the whole population (both males and females for all age groups)

We plotted the kernel densities per gender and age group and we added the national life expectancies trend line (red dashes) for comparison purposes and for each graph to describe better the estimated life expectancy. Figure 1 shows that the distribution of estimated life expectancy is not the same among the genders and age groups. Individuals in Dabou estimate their survival to be higher than the national average, and this is exacerbated for males (Table 2). However, for both males and females, and the three age groups considered herein, the distribution of estimated life expectancies appears to be a combination of at least two probability distributions. In each group, there is a proportion of individuals who had a low estimation of their life expectancy and those with medium or high estimations of their life expectancy. The same holds for the estimated global life expectancy distribution (Fig. 2). Several reasons, like changes in self-reported health [2] or perceived health conditions, might explain these differences.

**Table 2:** Comparison tests for estimated SLE and national average

Category	Estimated SLE	National average	Difference
Male 50–54	79.41 (0.58)	73.5	5.92^a^
Female 50–54	79.31 (1.04)	75.9	3.41^a^
Male 54–59	79.61 (0.65)	75.2	4.41^a^
Female 54–59	78.92 (1.20)	77.1	1.82
Male 60–64	79.91 (1.11)	77.4	2.51^b^
Female 60–64	79.30 (1.72)	78.7	0.60

Standard error in parenthesis

^a^significant at < 0.001

^b^significant at 5%

The red dashes for each sex and age group are the national life expectancy calculated using the actuarial method by the National Statistics Office in Côte d’Ivoire. The blue curve represents the kernel density of the estimated SLE. For males aged 50–54, the national life expectancy is 73.5 (red dashes, by actuarial method), while the kernel density shows that the major part of the male aged 50–54 has an estimated SLE higher than 73.5 (the density distribution is primarily located at the right side of the red dashes).

### Determinants of the self-assessed life expectancy

The determinants of the estimated life expectancy were modeled through a finite mixture of regression models. Based on the Akaike Information Criterion (AIC) and the log-likelihood, the model with four clusters/components was selected. Table 3 presents the AIC and log-likelihood values for the models with two, three, and four clusters/components.

**Table 3:** Information criterion for model selection

Number of components	AIC	Log-L
2	1440.088	− 674.0438
3	1453.651	− 653.8253
4	1426.568	− 613.284

*AIC* Akaike Information Criterion, *Log-L* Log-likelihood

The results of this selected model are presented in Table 4 and illustrate the effects of concomitant variables on the probability of being in each cluster and Table 5 for the determinants of the estimated life expectancy. Descriptive statistics about the clusters are given in Table 6. The average probabilities of being appropriately classified into each cluster are all above 80%, indicating that the model has good classification power. The first cluster represents 24.73% of the population, and the average SLE in this cluster is 79.51 years. The second cluster contains 20.97% of the sample. The average SLE of this cluster is quite similar to that of cluster 1 (78.89 years). That being said, compared to individuals in cluster 1, which reported a poor state of health reduces individual probability while having a mother alive or thinking that health limits the working capability increases the probability to be in cluster 2. Individuals in cluster 3 (33.33% of the sample) are characterized by deteriorating health and a chronic condition and the average SLE is the highest (80.02 years). Cluster 4 represents 20.97% of the population and is the cluster with the lowest average SLE (77.79 years). Having a chronic condition reduces an individual’s likelihood to be in cluster 4 while having a mother alive has the opposite effect. These results are consistent with the literature because individuals with a poor health state are pessimistic about their life expectancy [9]. Additionally, the literature confirms that having a parent alive increases individuals’ SLE [12]. However, we only found evidence that having a mother alive affects SLE. As for the determinants of SLE, there are some differences across clusters. In terms of demographic characteristics, couples have increased SLE for clusters 1 and 2, while having an opposite effect on cluster 4. Nonetheless, there was no significant effect of being a couple on the SLE of individuals in cluster 3. In both the highest (cluster 3 – optimistic individuals) and the lowest (cluster 4 – pessimistic individuals) SLE clusters, being male increases the SLE. However, this positive effect is reduced when the age increases. The opposite effect is observed for individuals in the two middle clusters (cluster 1 and 2 – moderate individuals) in which being a male decreases the SLE and this effect slows as age increases. Apart from cluster 1, where aging is negatively correlated with SLE, estimates reveal a positive and significant effect of aging on SLE. This finding is consistent with the results reported in the literature on the same topic [9]. Living in rural areas negatively affects the SLE for individuals for the two middle clusters (clusters 1 and 2). However, for both individuals with the lowest and highest estimates of SLE (clusters 3 and 4), living in rural areas increases the predicted life expectancy.

**Table 4:** Effects of concomitant variables on the probability to be classified in each cluster (component)

Concomitant variables	component 2^c^	component 3^c^	component 4^c^
Chronic condition	− 1.01 (0.63)	− 0.99^b^ (0.45)	−1.71^a^ (0.52)
Health condition limits working ability	2.16^b^ (0.87)	0.2 (0.52)	0.13 (0.58)
Deteriorating health	−0.37 (0.87)	−1.36^b^ (0.56)	−0.4 (0.54)
Poor health	−2.07^a^ (0.78)	0.16 (0.52)	0.56 (0.56)
Mother alive	1.32^b^ (0.63)	− 15.47 (781.08)	1.46^a^ (0.53)
Father alive	−3.49 (6.15)	−0.43 (0.97)	−16.16 (2722.68)
Both parents alive	−3.54 (11513)	16.22 (1585.7)	16.8 (1585.7)
Intercept	−0.09 (0.44)	0.89^b^ (0.38)	−0.12 (0.42)

Standard errors are in parenthesis

^a^significant at the 1% level

^b^significant at the 5% level

^c^Component 1 is the reference group

**Table 5:** Determinants of the estimated subjective life expectancy

Variables	Component 1 (SD) [24.73%]^d^	Component 2 (SD) [20.97%]^d^	Component 3 (SD) [33.33%]^d^	Component 4 (SD) [20.97%]^d^
Age	−0.3^a^ (0.04)	0.21^a^ (0.04)	0.45^a^ (0.06)	0.75^a^ (0.06)
Male	−48.45^a^ (4.19)	−6.18^b^ (2.64)	30.85^a^ (5.16)	11.65^a^ (4.15)
Male x Age	0.83^a^ (0.07)	0.12^b^ (0.05)	−0.54^a^ (0.09)	−0.15^b^ (0.07)
In a couple	0.65^b^ (0.28)	2.71^a^ (0.27)	−0.36 (0.41)	−1.75^a^ (0.52)
Employed	2.32^a^ (0.42)	5.23^a^ (0.29)	−0.78^c^ (0.45)	−5.23^a^(0.56)
Low school level	0.33 (0.3)	−0.88^a^ (0.3)	−2.51^a^ (0.43)	−3.46^a^ (0.43)
Income per capita	−0.38^b^ (0.15)	1.29^a^ (0.11)	−0.81^a^ (0.28)	1.11^a^ (0.27)
Squared income per capita	0.03^a^ (0.01)	−0.11^a^ (0.01)	0.05^b^(0.02)	−0.12^a^ (0.02)
Obese	−1.85^a^ (0.37)	2.95^a^ (0.2)	−1.75^a^ (0.48)	0.02 (0.36)
Current smoke	8.46^a^ (0.73)	−5.62^a^ (0.41)	−6.21^a^ (0.81)	4.91^a^ (0.58)
Alcohol consumption	−0.67^c^ (0.34)	−2.29^a^ (0.23)	−2.4^a^ (0.43)	2.45^a^ (0.36)
Does physical exercises	−2.05^a^ (0.31)	−2.48^a^ (0.25)	1.85^a^ (0.53)	4.56^a^ (0.49)
No diet	−2.86^a^ (0.28)	5.2^a^ (0.26)	2.39^a^ (0.38)	3.8^a^ (0.37)
Rural	−6.03^a^ (0.51)	−1.67^a^ (0.31)	1.48^a^ (0.55)	1.25^a^ (0.45)
Toilet with flush	1.72^a^ (0.33)	1.43^a^ (0.26)	−2.48^a^ (0.46)	1.6^a^ (0.47)
Underground water	−2.46^a^ (0.32)	7.98^a^ (0.25)	−2.42^a^ (0.44)	1.06^b^ (0.47)
Septic tank	−4.16^a^ (0.32)	2.88^a^ (0.31)	−0.63 (0.54)	5.22^a^ (0.55)
Intercept	105.15^a^ (2.69)	54.67^b^ (2.46)	60.01^a^ (3.84)	32.5^a^ (3.73)
Sigma	0.89	0.97	1.42	1.25

Standard errors

^a^significant at the 1% level

^b^significant at the 5% level

^c^significant at the 10% level

^d^Figures within brackets denote the proportion of respondents in the cluster/component

**Table 6:** Average values of key variables by clusters/components

Variables	Frequency (%)	Age (SD)	Expected life expectancy (SD)	Probability of classification (%)
Component 1	24*.*73	56 (0*.*52)	79*.*51 (0*.*48)	86*.*83
Component 2	20*.*97	57*.*02 (0*.*55)	78*.*89 (0*.*49)	80*.*32
Component 3	33*.*33	56*.*78 (0*.*46)	80*.*02 (0*.*36)	85*.*3
Component 4	20*.*97	55*.*71 (0*.*57)	77*.*79 (0*.*61)	90*.*56

*SD* Standard deviations

The effects of obesity also depend on the clusters. We did not find a significant association between obesity and SLE for individuals in the lowest cluster. However, for individuals in the highest two clusters of SLE, being obese reduces the SLE, while for those in cluster 2, obesity seems to increase their SLE. In terms of unhealthy behavior, we found that smoking reduces SLE for individuals in the highest cluster as well as those in cluster 2. However, for those in the lowest cluster and those in cluster 1, smoking has a positive effect on the SLE. This result has been explained in the literature as the optimism of smokers regarding their survival [16]. Excessive alcohol consumption also reduces SLE in all clusters, apart from individuals in the lowest cluster for whom it increases SLE. Rappange et al. also report that excessive alcohol consumption is positively associated with SSPs for some categories of individuals [13]. For individuals with the highest (cluster 3) and lowest (cluster 4) SLE, we found that performing physical activities increases individual SLE, while for those in the middle clusters (clusters 1 and 2), there is a decrease in SLE. Not having a specific diet increases SLE for individuals in all clusters, apart from those in the first middle cluster (cluster 1), for whom it reduces the SLE.

In terms of employment, we found that being employed reduces an individual’s SLE in both the highest and lowest SLE clusters, while the opposite effect was found for the two middle clusters. Having a low education/school level reduces an individual’s SLE in all clusters, apart from those in the first middle cluster, for which no significant effect was observed. This finding is consistent with the literature that highlights the heterogeneous effect of education on SLE [9, 17]. Estimates also show that the effect of income on an individual’s SLE is not linear. The effects of variable income on SLE are not linear across clusters. A U-shape effect is observed for individuals in the two highest clusters, while the opposite effect was found for those in the two lowest clusters. In other words, for those in the two highest clusters of SLE, an increase in household per capita income increased the SLE, but this increase abates when the household income per capita increases. The reverse effect is observed for those in the two lowest clusters. This result implies that for the pessimistic clusters, an increase in per capita income does not enhance the perceived life expectancy.

The effects of living standards on SLE are also very heterogeneous. Consuming underground water reduces SLE for those in the two highest SLE clusters and increases SLE for individuals in the two lowest clusters. Having toilets that flush increases the SLE for individuals in all clusters, apart from those in the highest cluster where the opposite effect is observed. Additionally, we found that having a septic tank increases SLE for the two lowest clusters, but reduces the SLE for those in cluster 1. For an individual in the highest cluster, no significant effect was found.

## Discussion

This manuscript analyzed the SLE among elders in Côte d’Ivoire. We proposed a novel approach to estimate the SLE. This approach builds on the method by Bellemare et al. that is based on splines [15]. It requires at least two points of SSPs to be assessed by individuals. Based on the estimated SLE, an analysis of its determinants is proposed. This analysis uses a finite mixture of the regression model to account for the heterogeneous distributions that compose the overall distribution of the SLE.

The estimated model allows for the classification of older people into clusters of people that have optimistic, pessimistic, and moderate self-assessment of their life expectancy. These clusters are discriminated against by the self-assessed poor state of health, the self-assessed decreasing health, the self-assessed working capacity limitation due to health, an observed chronic condition, and the mother being alive. In terms of determinants of SLE, the results suggest that the determinants of SLE vary among clusters. The pessimistic cluster is positively associated with poor health behavior (smoking, alcohol consumption, and having no diet). Results also suggest that low education levels and being employed are negatively associated with SLE while having a physical activity is positively associated with SLE for both pessimistic and optimistic clusters. Furthermore, a U-shape relationship was found between a household’s income per capita and SLE for the optimistic while a reverse U-shape relationship was found for the pessimistic. The role of socioeconomic and living condition variables has also been highlighted.

It is worth noting that our results are in line with existing literature dealing with different aspects of SLE [9, 12, 16, 17]. These results suggest that promoting a healthy lifestyle, improving health and living conditions, as well as employment status, are key to increasing elders’ SLE. However, even though the results are promising, they lack generalizability. The dataset used covers only one region of the country. Furthermore, due to its geographic proximity (under 50 km and accessible by paved road) with the largest urban center of the country (the economic capital, Abidjan), this region benefits from the health facilities provided in the capital. This might affect individuals’ perception of their health and this region could differ from others. Finally, Subjective expectations of outcomes such as life expectancy depend on the subject’s psychological profile or personality traits. However, we did not collect information on the subject’s psychological profile or personal traits and therefore did not adjust for them in our analyses.

## Conclusion

In this paper, we combined a spline-based approach and a finite mixture of regression models to estimate the SLE based on the SSPs and identified the determinants of the SLE among elders, respectively. Doing so, we improved the estimation of subjective life expectancy by accounting for the heterogeneity of the distribution of the estimated subjective life expectancy. This approach is an improvement of the usual practices for estimating individual subjective life expectancy and may provide insights into the elderly’s perception of aging, which could be used to forecast the demand for health services and long-term care needs. It may also provide important information for the development of health policy strategies aimed at addressing the determinants of subjective life expectancy. Further studies on the estimation of subjective life expectancy in developing countries, Côte d’Ivoire for instance, are warranted.

## Supplementary information

**Additional file 1.** This supplementary file is our study questionnaire 1 titled “The living condition, resilience, and health among the elderly survey – Household section”, which has been cited in the main manuscript - page 7, under the sub-heading, Data of the manuscript.

**Additional file 2.** This supplementary file is our study questionnaire 2 titled “The living condition, resilience, and health among the elderly survey – Health section”, which has been cited in the main manuscript - page 7, under the sub-heading, “Data” of the manuscript.

**Additional file 3.** This supplementary file is a technical note on the methods used in this paper and cited in the main manuscript – page 9, under the sub-heading, “Estimation of self-assessed life expectancy” of the manuscript.

## Data Availability

The datasets generated during and/or analyzed during the current study are available in the Mendeley repository, https://data.mendeley.com/datasets/28r5wwvwt7/2

## Abbreviations

SLE: Subjective life expectancy
HIV/AIDS: Human Immunodeficiency Virus/Acquired Immune Deficiency Syndrome
SSPs: Self-assessed survival probabilities
ENSEA: Ecole Nationale Supérieure de Statistique et d’Economie Appliquée
