# Supplementary material for: Self-assessed life expectancy among older adults in Côte d’Ivoire
Source: BMC Public Health. 2020 Jun 15;20:932. doi: 10.1186/s12889-020-09034-4 (PMC7296699; doi:10.1186/s12889-020-09034-4)
Supplement: Supplementary file 1 — Additional file 1. This supplementary file is our study questionnaire 1 titled “The living condition, resilience, and health among the elderly survey – Household section”, which has been cited in the main manuscript - page 7, under the sub-heading, Data of the manuscript. [file 12889_2020_9034_MOESM1_ESM.docx]

**IDENTIFICATION**

| **I01** DEPARTMENT------------------------------------------**\|___\|___\|**  **I02**LOCALITY -----------------------------------------------**\|___\|___\|**  **I03** ENUMERATION AREA ----------**\|___\|___\|___\|___\|**  **I04** SUB ENUMERATION AREA**---------------------------\|___\|___\|___\|___\|** | **I05** BULDING NUMBER ---------------------**\|___\|___\|___\|**  **I06** HOUSEHOLD NUMBER-----------------------**\|___\|___\|___\|**  **I07** AREA OF RESIDENCE-------------------------------------**\|___\|**  1 = Rural  2 = Urban  **I08** HOUSEHOLD QUESTIONNAIRE ID ……/……. |
| --- | --- |

| SUPERVISOR NAME ------------------------------------------------------------------- SUPERVISOR ID CODE ----------**\|___\|___\|___\|**  ENUMERATOR NAME-------------------------------------------------------------------ENUMERATOR ID CODE-----------**\|___\|___\|___\|**  INTERVIEW DATE------ **\|___\|___\|\|___\|___\| \|__\|__\|__\|__\|**  START TIME--------H--------- END TIME--------H--------- |
| --- |

**SUMMARY OF HOUSEHOLD COMPOSITION**

| GENDER | RESIDENTS | | | VISITORS | TOTAL |
| --- | --- | --- | --- | --- | --- |
|  | Present | Absent | Total |  |  |
| MALE |  |  |  |  |  |
| FEMALE |  |  |  |  |  |
| TOTAL |  |  |  |  |  |

CONFIDENTIALITY: The information collected through this questionnaire is strictly confidential. They are under statistical secrecy and could not be published without anonymization concerning the 2013’s law on statistical secrecy.

**SECTION A: HOUSEHOLD MEMBERS CHARACTERISTICS I**

| **ID** | **NAME** | **RELATION WITH HEAD** | **RESIDENTIAL STATUS** | **GENDER** | **BIRTHDATE** | **CIVIL STATUS** | **COUNTRY/CITY OF BIRTH** | | **RELIGION** | **ETHNICITY** |
| --- | --- | --- | --- | --- | --- | --- | --- | --- | --- | --- |
|  |  | 01- Head  02- Spouse  03- Son / daughter  04-Father / mother  05- Brother/sister  06- Grandson  07- Grandparent  08- Other parents  09- Stepparents  10- Unrelated | Does (NAME) usually live here?  Does (NANE) passed last night here?  1- Present  2- Absent  3-Visitor | 1=Male  2=Female | Enter the date of birth following the format **(DD/MM/YY)**  **Or enter the AGE**  If age is entered, specify the unit using:  1 = day  2 = month  3 = year | Is (NAME) registered at the civil status register?  1 = Yes  0 = No | Is (NAME) born in Côte d’Ivoire?  1= Yes  0= No  If A06A=1 skip to A07 | Where were (NAME) born?  1=ECOWAS country  2=Country out of ECOWAS  (specify the country) | 1 = CATHOLIC  2 = PROTESTANT  3 = OTHER CHRISTIAN  4 = MUSLIM  5 = AFRICAN RELIGION  6 = OTHER RELIGIONS  7= NO RELIGION | Ethnicity if Ivorian,  Nationality for others |
| **I09** | **A01** | **A02** | **A03** | **A04** | **A05A** | **A05B** | **A06A** | **A06B** | **A07** | **A08** |
| **\|__\|__\|** |  | **\|__\|__\|** | **\|__\|** | **\|__\|** | **\|__\|__\| / \|__\|__\| / \|__\|__\|__\|__\|**  **\|__\|__\| / __\|** | \|__\| | **\|__\|** | **\|__\|** | **\|__\|** | **\|__\|__\|** |
| **\|__\|__\|** |  | **\|__\|__\|** | **\|__\|** | **\|__\|** | **\|__\|__\| / \|__\|__\| / \|__\|__\|__\|__\|**  **\|__\|__\| / __\|** | \|__\| | **\|__\|** | **\|__\|** | **\|__\|** | **\|__\|__\|** |
| **\|__\|__\|** |  | **\|__\|__\|** | **\|__\|** | **\|__\|** | **\|__\|__\| / \|__\|__\| / \|__\|__\|__\|__\|**  **\|__\|__\| / __\|** | \|__\| | **\|__\|** | **\|__\|** | **\|__\|** | **\|__\|__\|** |
| **\|__\|__\|** |  | **\|__\|__\|** | **\|__\|** | **\|__\|** | **\|__\|__\| / \|__\|__\| / \|__\|__\|__\|__\|**  **\|__\|__\| / __\|** | \|__\| | **\|__\|** | **\|__\|** | **\|__\|** | **\|__\|__\|** |
| **\|__\|__\|** |  | **\|__\|__\|** | **\|__\|** | **\|__\|** | **\|__\|__\| / \|__\|__\| / \|__\|__\|__\|__\|**  **\|__\|__\| / __\|** | \|__\| | **\|__\|** | **\|__\|** | **\|__\|** | **\|__\|__\|** |
| **\|__\|__\|** |  | **\|__\|__\|** | **\|__\|** | **\|__\|** | **\|__\|__\| / \|__\|__\| / \|__\|__\|__\|__\|**  **\|__\|__\| / __\|** | \|__\| | **\|__\|** | **\|__\|** | **\|__\|** | **\|__\|__\|** |
| **\|__\|__\|** |  | **\|__\|__\|** | **\|__\|** | **\|__\|** | **\|__\|__\| / \|__\|__\| / \|__\|__\|__\|__\|**  **\|__\|__\| / __\|** | \|__\| | **\|__\|** | **\|__\|** | **\|__\|** | **\|__\|__\|** |
| **\|__\|__\|** |  | **\|__\|__\|** | **\|__\|** | **\|__\|** | **\|__\|__\| / \|__\|__\| / \|__\|__\|__\|__\|**  **\|__\|__\| / __\|** | \|__\| | **\|__\|** | **\|__\|** | **\|__\|** | **\|__\|__\|** |
| **\|__\|__\|** |  | **\|__\|__\|** | **\|__\|** | **\|__\|** | **\|__\|__\| / \|__\|__\| / \|__\|__\|__\|__\|**  **\|__\|__\| / __\|** | \|__\| | **\|__\|** | **\|__\|** | **\|__\|** | **\|__\|__\|** |
| **\|__\|__\|** |  | **\|__\|__\|** | **\|__\|** | **\|__\|** | **\|__\|__\| / \|__\|__\| / \|__\|__\|__\|__\|**  **\|__\|__\| / __\|** | \|__\| | **\|__\|** | **\|__\|** | **\|__\|** | **\|__\|__\|** |

**SECTION A: HOUSEHOLD MEMBERS CHARACTERISTICS II**

| Aged 3 and above | | | aged 3- 24 | | | | | Aged 5 and above | | | Aged 12 and above |
| --- | --- | --- | --- | --- | --- | --- | --- | --- | --- | --- | --- |
| Able to read and write in any language? | **(NAME) ever go to school** | **What is the school level of (NAME)** | **Did (NAME) go to school last year (2015 /2016)** | **If yes, at what level (classroom)?** | **Did (NAME) go to school this year (2016 /2017)** | **If yes, at what level (classroom)?** | **HANDICAP** | **Occupation status** | **Main job** | **Job status** | **Marital status** |
| A09 | **A10** | **A11** | **A12** | **A13** | **A14** | **A15** | **A16** | **A17** | **A18** | **A19** | **A20** |
| (show any text to the surveyed and let him/her read and write it)  1-reading only  2–writing only  3-none of reading and writing  4-both reading and writing | 1 = Yes  0 = No  If 0 skip to A16 | 0 = kindergarten  1 = Islamic school  2 = primary school  3 = secondary school  4 = high school  5 = university | 1 = Yes  0 = No  ***If A12=0 skip to A14*** | 0= kindergarten  1= 1^st^ primary  2=2^nd^ primary  3=3^rd^ primary  4=4^th^ primary  5=5^th^ primary  6=6^th^ primary  7=1^st^ sec.  8=2^nd^ sec.  9=3^rd^ sec.  10=4^th^ sec.  11=1^st^ high sc.  12=2^nd^ high sc.  13=3^rd^ high sc.  14 = 1^st^ univ.  15= 2^nd^ univ  16=3^rd^ univ  17= 4^th^ univ  18= 5^th^ univ + | 1 = Yes  0 = No  ***If A14=0 skip to A16*** | 0= kindergarten  1= 1^st^ primary  2=2^nd^ primary  3=3^rd^ primary  4=4^th^ primary  5=5^th^ primary  6=6^th^ primary  7=1^st^ sec.  8=2^nd^ sec.  9=3^rd^ sec.  10=4^th^ sec.  11=1^st^ high sc.  12=2^nd^ high sc.  13=3^rd^ high sc.  14 = 1^st^ univ.  15= 2^nd^ univ  16=3^rd^ univ  17= 4^th^ univ  18= 5^th^ univ + | A=without Handicap  B=blinded  C=deaf  D=mute  E=stutterer  F=albino  G=Handicap of legs  I= mental handicap  J=others physical handicaps | 1 = occupied  2 = unemployed  3 = looking for first job  4 = housewife  5 = student  6 = retired  7 = other inactive  If not occupied, skip to A20 | Describe the occupation  (Only for those whom are occupied) | 1=Employer  2=Salaried  3=Independent  4=Caregiver  5= Apprentice  6=task worker  8=other | 1 = Never married  2 = married  3 = married (polygamous)  4 = divorced  5 = windowed  6 = in couple (without marriage) |
| \|__\| | **\|__\|** | **\|__\|** | **\|__\|** | **\|__\|__\|** | **\|__\|** | **\|__\|__\|** | **\|__\|__\|__\|** | **\|__\|** | ……………………………… | **\|__\|** | **\|__\|** |
| \|__\| | **\|__\|** | **\|__\|** | **\|__\|** | **\|__\|__\|** | **\|__\|** | **\|__\|__\|** | **\|__\|__\|__\|** | **\|__\|** | ……………………………… | **\|__\|** | **\|__\|** |
| \|__\| | **\|__\|** | **\|__\|** | **\|__\|** | **\|__\|__\|** | **\|__\|** | **\|__\|__\|** | **\|__\|__\|__\|** | **\|__\|** | ……………………………… | **\|__\|** | **\|__\|** |
| \|__\| | **\|__\|** | **\|__\|** | **\|__\|** | **\|__\|__\|** | **\|__\|** | **\|__\|__\|** | **\|__\|__\|__\|** | **\|__\|** | ……………………………… | **\|__\|** | **\|__\|** |
| \|__\| | **\|__\|** | **\|__\|** | **\|__\|** | **\|__\|__\|** | **\|__\|** | **\|__\|__\|** | **\|__\|__\|__\|** | **\|__\|** | ……………………………… | **\|__\|** | **\|__\|** |
| \|__\| | **\|__\|** | **\|__\|** | **\|__\|** | **\|__\|__\|** | **\|__\|** | **\|__\|__\|** | **\|__\|__\|__\|** | **\|__\|** | ……………………………… | **\|__\|** | **\|__\|** |
| \|__\| | **\|__\|** | **\|__\|** | **\|__\|** | **\|__\|__\|** | **\|__\|** | **\|__\|__\|** | **\|__\|__\|__\|** | **\|__\|** | ……………………………… | **\|__\|** | **\|__\|** |
| \|__\| | **\|__\|** | **\|__\|** | **\|__\|** | **\|__\|__\|** | **\|__\|** | **\|__\|__\|** | **\|__\|__\|__\|** | **\|__\|** | ……………………………… | **\|__\|** | **\|__\|** |
| \|__\| | **\|__\|** | **\|__\|** | **\|__\|** | **\|__\|__\|** | **\|__\|** | **\|__\|__\|** | **\|__\|__\|__\|** | **\|__\|** | ……………………………… | **\|__\|** | **\|__\|** |
| \|__\| | **\|__\|** | **\|__\|** | **\|__\|** | **\|__\|__\|** | **\|__\|** | **\|__\|__\|** | **\|__\|__\|__\|** | **\|__\|** | …………………………………… | **\|__\|** | **\|__\|** |
| \|__\| | **\|__\|** | **\|__\|** | **\|__\|** | **\|__\|__\|** | **\|__\|** | **\|__\|__\|** | **\|__\|__\|__\|** | **\|__\|** | …………………………………… | **\|__\|** | **\|__\|** |

Sec: Secondary; Sc: School; Univ: University

**SECTION A: HOUSEHOLD MEMBERS CHARACTERISTICS III**

| **NUMERO D’ORDRE** | **(NAME)’s parents are alive?**  **if A21=0 skip to A25**  **if A21=2 skip to A23** | **If the mother is alive,**  **What is her school level?** | **If the father is alive,**  **What is his school level?** | **If at least one of the parents is alive, does he lives in the same household?** | **Has (NAME) ever live in another country for at least 6 months?** | **Has (NAME) seen a health professional during the last 4 weeks?** | **What kind of health professional (NAME) consulted during the last 4 weeks?** | **What was the reason for the consultation?** | **How many consultations did (NAME) have in the last 4 weeks?** | **Where was the last consultation?** |
| --- | --- | --- | --- | --- | --- | --- | --- | --- | --- | --- |
|  | **A21** | **A22** | **A23** | **A24** | **A25** | **A26** | **A27** | **A28** | **A29** | **A30** |
|  | 0-none of them  1-mother only  2-father only  3-both parents alive | 0 = kindergarten  1 = Koranic  2 = primary school  3 = secondary school  4 = high school  5 = university | 0 = kindergarten  1 = Koranic  2 = primary school  3 = secondary school  4 = high school  5 = university | 0-none of them  1-mother only  2-father only  3-both parents | 1-Yes  0-No | 1-Yes  0-No  if A26=0 skip to A02 (next household member) | 1- ASC  2-medical assistant  3-nurse  4-doctor  5- pharmacist  6- other | 01-Fever/malaria  02- Diarrhoea  03-Stomach upset  04-vomiting  05- sore throat  06 nasal breaths (sinus)  07-pulmonary respiration  08- Flu  09- asthma  10- headache  11-fainting  12- skin problems  13-dental problems  14-eye problems  15- ENT  16-Consultations related to pregnancy  17-Others (to be specified) |  | 1-health hut  2-health centre  3-general hospital  4-Regional hospital centre  5-University hospital centre  6- specialized centre  7- private clinical  8- at home  9- other |
| **\|__\|__\|** | **\|__\|** | **\|__\|** | **\|__\|** | **\|__\|** | **\|__\|** | **\|__\|** | **\|__\|__\|** | **\|__\|__\|** | **\|__\|__\|** | **\|__\|** |
| **\|__\|__\|** | **\|__\|** | **\|__\|** | **\|__\|** | **\|__\|** | **\|__\|** | **\|__\|** | **\|__\|__\|** | **\|__\|__\|** | **\|__\|__\|** | **\|__\|** |
| **\|__\|__\|** | **\|__\|** | **\|__\|** | **\|__\|** | **\|__\|** | **\|__\|** | **\|__\|** | **\|__\|__\|** | **\|__\|__\|** | **\|__\|__\|** | **\|__\|** |
| **\|__\|__\|** | **\|__\|** | **\|__\|** | **\|__\|** | **\|__\|** | **\|__\|** | **\|__\|__\|** | **\|__\|__\|** | **\|__\|__\|** | **\|__\|** | **\|__\|__\|** |
| **\|__\|__\|** | **\|__\|** | **\|__\|** | **\|__\|** | **\|__\|** | **\|__\|** | **\|__\|__\|** | **\|__\|__\|** | **\|__\|__\|** | **\|__\|** | **\|__\|__\|** |
| **\|__\|__\|** | **\|__\|** | **\|__\|** | **\|__\|** | **\|__\|** | **\|__\|** | **\|__\|__\|** | **\|__\|__\|** | **\|__\|__\|** | **\|__\|** | **\|__\|__\|** |
| **\|__\|__\|** | **\|__\|** | **\|__\|** | **\|__\|** | **\|__\|** | **\|__\|** | **\|__\|__\|** | **\|__\|__\|** | **\|__\|__\|** | **\|__\|** | **\|__\|__\|** |
| **\|__\|__\|** | **\|__\|** | **\|__\|** | **\|__\|** | **\|__\|** | **\|__\|** | **\|__\|__\|** | **\|__\|__\|** | **\|__\|__\|** | **\|__\|** | **\|__\|__\|** |
| **\|__\|__\|** | **\|__\|** | **\|__\|** | **\|__\|** | **\|__\|** |  | **\|__\|__\|** | **\|__\|__\|** | **\|__\|__\|** | **\|__\|** | **\|__\|__\|** |
| **\|__\|__\|** | **\|__\|** | **\|__\|** | **\|__\|** | **\|__\|** | **\|__\|** | **\|__\|__\|** | **\|__\|__\|** | **\|__\|__\|** | **\|__\|** | **\|__\|__\|** |
| **\|__\|__\|** | **\|__\|** | **\|__\|** | **\|__\|** | **\|__\|** | **\|__\|** | **\|__\|__\|** | **\|__\|__\|** | **\|__\|__\|** | **\|__\|** | **\|__\|__\|** |

**SECTION B: HOUSING CHARACTERISTICS**

| **Dwelling occupational status** | **Monthly rent (XOF)** | **Number of rooms** | **Used room** | **Type of dwelling** | **Main type of toilet** | **Main water source** | **Main lighting source** |
| --- | --- | --- | --- | --- | --- | --- | --- |
| **B01** | **B02** | **B03** | **B04** | **B05** | **B06** | **B07** | **B08** |
| 1=owner  2=familial dwelling  3 = Tenant  4 = Official house / for free  5 = Official house / Package  8 = Other (to be specified) | If you are a tenant “How much do you pay your rent per month? "  If not "How much do you plan to rent the accommodation to someone else"? | How many rooms in your dwelling? | How many rooms do you use for sleeping? | 1 = Villa  2 = Detached house  3 = Tape slot  4 = Apartment in a building  5 = Concession / common court  6 = Traditional box  7 = Barracks  8 = Other (to be specified)  ……………….. | 1 = Toilets with flush  2 = Toilets without flushing  3 = Latrine in the courtyard  4 = Latrines out of the yard  5 = In nature  8 = Other (to be specified) | 1 = sate water (SODECI)  2 = Village pump  3 = good  4 = Source, river, backwater  8 = Other (to be specified) | 1 = grid electricity  2 = private electricity (group, solar panel)  3 = Gas lamp  4 = flashlight  5 = kerosene lamp  6 = Wood fire  8 = Other (to be specified) |
| **\|___\|** | \|__\|__\|__\|__\|__\|__\| | **\|___\|___\|** | **\|___\|___\|** | **\|___\|** | **\|___\|** | **\|___\|** | **\|___\|** |

| **Main source of energy for cooking** | **Main household waste disposal method** | **Main wastewater disposal method** | **Equipment for mobility** | **Household appliances** | **Audio-visual equipment and TIC** |
| --- | --- | --- | --- | --- | --- |
| **B09** | **B10** | **B11** | **B12** | **B13** | **B14** |
| 1 = wood  2 = charcoal  3 = Gas  4 = electricity  5 = manure  8 = Other (to be specified) | 1 = Private collection / Town hall  2 = Piers in nature  3 = Jetties in the street / ditch  4 = buried  5 = burned  6 = recycled  8 = Other (to be specified) | 1 = courtyard  2 = street  3 = channel  4 = Septic tank  8 = Other (to be specified) | A = Bike \| __ \|  B = motorcycle \| __ \|  C = tricycle \| __ \|  D = vehicle \| __ \|  E = canoe \| __ \|  F = carriage \| __ \|  X= Other (to be specified) **\|__\|**  **…………………………………..**…………………. | A = fan \| __ \|  B = Refrigerator \| __ \|  C = Gas stove . \| __ \|  D = iron \| __ \|  E = Air conditioner \| __ \|  X= Other (to be specified) **\| __ \|**  …………………………………………………………………………………………..…………………. | A=Radio **\|__\|**  B=TV **\|__\|**  C=Mobile phone **\|__\|**  D= Phone **\|__\|**  E=Computer **\|__\|**  F= Internet connexion **\|__\|**  X=Other (to be specified) **\|__\|**  **……………………………………………………………….** |
| **\|___\|** | **\|___\|** | **\|___\|** | \|__\|__\|__\|__\|__\|__\|__\| | \|__\|__\|__\|__\|__\|__\| | \|__\|__\|__\|__\|__\|__\|__\| |

**SECTION C: HOUSEHOLD INCOME AND EXPENDITURES**

| **Who is in charge of … expenditures in the household?** | | | | | | |
| --- | --- | --- | --- | --- | --- | --- |
| **Health** | **Food** | **Transport** | **Communication** | **Equipment** | **Electricity and water** | **Education** |
| **C01** | **C02** | **C03** | **C04** | **C05** | **C06** | **C07** |
| A = Head of household  B = Household member other than the head  C = Health insurance  D = External support  X = Other (to be specified)……………… | A = Head of household  B = Household member other than the Head  C = External support  X = Other (to be specified)…………………………………………… | A = Head of household  B = Household member other than the Head  C = External support  X = Other (to be specified)…………………………………… | A = Head of household  B = Household member other than the Head  C = External support  X = Other (to be specified)………………………………………… | A = Head of household  B = Household member other than the Head  C = External support  X = Other (to be specified)………………………………………… | A = Head of household  B = Household member other than the Head  C = External support  X = Other (to be specified)………………………………………… | A = Head of household  B = Household member other than the Head  C = External support  X = Other (to be specified)……………………………………… |
| **Average monthly (XOF) expenses of your household in** | | | | | | |
| **Health** | **Food** | **Transport** | **Communication** | **Equipment** | **Electricity and water** | **Education** |
| **C08** | **C09** | **C10** | **C11** | **C12** | **C13** | **C14** |
| \|__\|__\|__\|__\|__\|__\| | \|__\|__\|__\|__\|__\|__\| | \|__\|__\|__\|__\|__\|__\| | \|__\|__\|__\|__\|__\|__\| | \|__\|__\|__\|__\|__\|__\| | \|__\|__\|__\|__\|__\|__\| | \|__\|__\|__\|__\|__\|__\| |

| **Who is in charge of the expenditures in the household?** | | | **Who provides income for the household?** | | | |
| --- | --- | --- | --- | --- | --- | --- |
| **Clothing** | **Leisure** | **Other** | **Main job income** | **Secondary job income** | **Monetary remittance** | **Transfers in kind** |
| **C15** | **C16** | **C17** | **C18** | **C19** | **C20** | **C21** |
| A = Head of household  B = Household member other than the Head  C = External support  X = Other (to be specified)  ……………………………………………………. | A = Head of household  B = Household member other than the Head  C = External support  X = Other (to be specified)…………………………………………………… | A = Head of household  B = Household member other than the Head  C = External support  X = Other (to be specified)  …………………………………………………… | A = Head of household  B = Household member other than the Head  C = External support  X = Other (to be specified)…………………………………………………… | A = Head of household  B = Household member other than the Head  C = External support  X = Other (to be specified)……………………………………… | A = Head of household  B = Household member other than the Head  C = External support  X = Other (to be specified)…………………………………………………… | A = Head of household  B = Household member other than the Head  C = External support  X = Other (to be specified)  …………………………………………… |
| **Average monthly expenses of your household in terms of** | | | **Average monthly income** | | | |
| **Clothing** | **Leisure** | **Other** | **Main job income** | **Secondary job income** | **Monetary remittance** | **Transfers in kind** |
| **C22** | **C23** | **C24** | **C25** | **C26** | **C27** | **C28** |
| \|__\|__\|__\|__\|__\|__\| | \|__\|__\|__\|__\|__\|__\|__ | \|__\|__\|__\|__\|__\|__\|__ | \|__\|__\|__\|__\|__\|__\|__ | \|__\|__\|__\|__\|__\|__\|_ | \|__\|__\|__\|__\|__\|__\|__ | \|__\|__\|__\|__\|__\|__\| |

***THANK YOU FOR YOUR KIND COLLABORATION***
