# Supplementary material for: Self-assessed life expectancy among older adults in Côte d’Ivoire
Source: BMC Public Health. 2020 Jun 15;20:932. doi: 10.1186/s12889-020-09034-4 (PMC7296699; doi:10.1186/s12889-020-09034-4)
Supplement: Supplementary file 2 — Additional file 2. This supplementary file is our study questionnaire 2 titled “The living condition, resilience, and health among the elderly survey – Health section”, which has been cited in the main manuscript - page 7, under the sub-heading, “Data” of the manuscript. [file 12889_2020_9034_MOESM2_ESM.docx]

**IDENTIFICATION**

| I1 – Department /__/__/ | I5 – Household number /__/__/__/ |
| --- | --- |
| I2 – Locality /__/__/ | I6 – Residence area (1-Urban ; 2- Rural) /__/ |
| I3 – Enumeration Area /__/__/__/__/ | I7 – household questionnaire ID /__/__/__/ |
| I4 – Sub Enumeration area /__/__/__/__/ | I8 – ID of the respondent with household /__/__/ |
| Interview date Day /__/__/ Month /__/__/ Year /__/__/__/__/  Start time /__/__/ /__/__/ End time /__/__/ /__/__/  Enumerator ID code /__/__/__/__/ Supervisor ID code /__/__/__/__/ | |

**Section I: Physical health**

| **Code** | **Questions and answers options** | **Responses** |
| --- | --- | --- |
| SP1 | How tall are you? (answer in centimetre) | /__/__/__/ |
| SP2 | What is your weight? (answer in kilogram) | /__/__/__/ |
| SP3 | How is your hearing?  **(1- Excellent, 2- Very good, 3 - Good, 4 - Fair, 5- Poor)** | /__/ |
| SP4 | How is your eyesight?  **(1- Excellent, 2- Very good, 3 - Good, 4 - Fair, 5- Poor)** | /__/ |
| SP5 | How is your health in general?  **(1- Excellent, 2- Very good, 3 - Good, 4 - Fair, 5- Poor)** | /__/ |
| SP6 | How was your health in general last year?  **(1- Excellent, 2- Very good, 3 - Good, 4 - Fair, 5- Poor)** | /__/ |
| SP7 | Do you have a chronic illness?  **(1- Yes, 0- No)** | /__/ |
| SP8 | Does your state of health limit your ability to do your activities?  **(1- Severely limited, 2- Limited, but not severely, 3- Not limited)** | /__/ |
| SP9 | Did your doctor tell you had one of the conditions below **(1- Yes, 0- No)?** If yes, when?   1. Heart attack 2. High blood pressure or hypertension 3. High blood cholesterol 4. Stroke 5. Diabetes or high blood sugar 6. Chronic lung disease 7. Asthma 8. Arthritis 9. Osteoporosis 10. Cancer 11. Stomach or duodenal ulcer, peptic ulcer 12. Parkinson disease 13. Cataract 14. Tumour 15. Sickle cell anemia 16. Other (provide precision) ---------------------------------- 17. None of the above mentioned | /__/  /__/ date /__/__/__/__/  /__/ date /__/__/__/__/  /__/ date /__/__/__/__/  /__/ date /__/__/__/__/  /__/ date /__/__/__/__/  /__/ date /__/__/__/__/  /__/ date /__/__/__/__/  /__/ date /__/__/__/__/  /__/ date /__/__/__/__/  /__/ date /__/__/__/__/  /__/ date /__/__/__/__/  /__/ date /__/__/__/__/  /__/ date /__/__/__/__/  /__/ date /__/__/__/__/  /__/ date /__/__/__/__/  /__/ date /__/__/__/__/  /__/ |
| SP10 | Are you treated for **(1- Yes, 0- No)**   1. high blood pressure or hypertension? 2. hypertension? 3. other heart diseases? 4. asthma? 5. diabetes or high blood sugar? 6. joint pain? 7. sleep disorder? 8. anxiety or depression? 9. heartburn? 10. chronic bronchitis? 11. other diseases? Please specify------------------------------------------- 12. no ongoing treatment? | /__/  /__/  /__/  /__/  /__/  /__/  /__/  /__/  /__/  /__/  /__/  /__/ |
| SP11 | Are you currently smoking? **(1- Yes, 0- No) If yes, skip to SP15** | /__/ |
| SP12 | Have you ever smoked?  **(1- Yes, 0- No)** | /__/ |
| SP13 | If yes to SP12, since when have you stopped smoking? | date /__/__/__/__/ |
| SP14 | If currently smoker, how often do you smoke?  **1- Every day; 2- Usually; 3- Rarely** | /__/ |
| SP15 | Do you drink alcohol (more than two glasses)?  **(1- Yes, 0- No)** | /__/ |
| SP16 | If yes, how often do you drink alcohol?  **(1- Every day; 2- Once a week; 3- Twice per week); 4- Rarely** | /__/ |
| SP17 | Do you have difficulties in **(1- Not at all; 2- Sometimes; 3- Usually; 4- Always)**:   1. walking long distances (more than 1 kilometre for example)? 2. walking small distances (100 meters for example)? 3. sitting for two hours? 4. getting up from a chair? 5. climbing several flights of stairs? 6. climbing one flight of stairs? 7. stooping, kneeling, crouching? 8. reaching or extending your arms above your shoulders? 9. pulling or pushing large objects? 10. lifting or carrying weights over 5 kilograms? 11. picking up a small coin from a table? 12. None of the above mentioned | /__/  /__/  /__/  /__/  /__/  /__/  /__/  /__/  /__/  /__/  /__/ |
| SP18 | Do you need assistance for:  **(1- Not at all; 2- Sometimes; 3- Usually; 4- Always)**   1. eating? 2. dressing? 3. bathing? 4. using toilets? 5. traveling/moving from a place to another? | /__/  /__/  /__/  /__/  /__/ |
| SP19 | Do you have to stay in bed or sit all day?  **(1- Not at all; 2- Sometimes; 3- Usually; 4- Always)** | /__/ |
| SP20 | During the last week: **(1- Not at all; 2- Sometimes; 3- Usually; 4- Always)**   1. did you have difficulty carrying out your daily work or tasks? 2. did you have difficulty with your hobbies or leisure activities? 3. have you been out of breath? 4. have you had any pain? 5. did you need to rest? 6. have you had any sleep disturbances? 7. did you feel weak? 8. did you have a lack of appetite? 9. have you had nausea? 10. did you vomit? 11. have you been constipated? 12. have you had diarrhea? 13. were you tired? 14. did the pains upset your daily activities? 15. did you have difficulty concentrating (reading or watching tv for example)? 16. did you feel tense? 17. were you anxious? 18. did you feel irritable? 19. did you feel depressed? 20. did you have difficulty remembering things 21. did your physical condition or your medical treatment alter your family life? 22. did your physical condition or your medical treatment alter your social activities? 23. did your physical condition or medical treatment cause you financial difficulties? | /__/  /__/  /__/  /__/  /__/  /__/  /__/  /__/  /__/  /__/  /__/  /__/  /__/  /__/  /__/  /__/  /__/  /__/  /__/  /__/  /__/  /__/  /__/ |
| SP21 | How was your general health last week? (scale from 1 to 7)  **(1- Excellent et 7- pour)** | /__/ |
| SP22 | How was your quality of life during last week? (scale from 1 to 7)  **(1- Excellent et 7- poor)** | /__/ |
| SP23 | On a scale of 0 to 100, how much do you estimate your chances of being alive at target age:   1. 70 2. 75 3. 80 4. 85 | /__/__/__/  /__/__/__/  /__/__/__/  /__/__/__/ |

**Section II: health behavior and health services use**

| **Code** | **Questions and answers options** | **Responses** |
| --- | --- | --- |
| CS1 | Have you been hospitalized in the last 12 months?  **(1- Yes, 0- No)** | /__/ |
| CS2 | If yes, how many times? | /__/__/ |
| CS3 | Have you seen a general practitioner in the last 12 months?  **(1- Yes, 0- No)** | /__/ |
| CS4 | If yes, how many times? | /__/__/ |
| CS5 | Have you seen a specialist doctor in the last 12 months?  **(1- Yes, 0- No)** | /__/ |
| CS6 | If yes, how many times did you see a(n)   1. ophthalmologist? 2. dentist? 3. dermatologist? 4. gynecologist? 5. ENT doctor (Ear, Nose, Throat)? 6. other ……………… | /__/__/  /__/__/  /__/__/  /__/__/  /__/__/  /__/__/ |
| CS7 | Have you used self-medication in the past 12 months?  **(1- Yes, 0- No)** | /__/ |
| CS8 | If yes, what was the reason?.................................................................... Quantify the severity of the reason: **1 Very serious; 2-Serious; 3- Not serious;** | /__/ |
| CS9 | Have you consulted a traditional practitioner in the past 12 months? **(1- Yes, 0- No)** | /__/ |
| CS10 | If yes, what was the reason?.................................................................... Quantify the severity of the reason: **1 Very serious; 2-Serious; 3- Not serious;** | /__/ |
| CS11 | Do you exercise (sport)?  **1- Yes, 0- No** | /__/ |
| CS12 | If yes, how often?  **1-every day; 2-many times per week; 3- once per week; 4-rarely** | /__/ |
| CS13 | Do you have any diet? **(1- Yes, 0- No)**   1. No fatty foods 2. No salty foods 3. No sweet food 4. Other _________________________________ 5. None | /__/  /__/  /__/  /__/  /__/ |
| CS14 | Did a doctor suggest this diet? **(1- Yes, 0- No)** | /__/ |

**Section III: health expenditures**

| **Code** | **Questions and answers options** | **Responses** |
| --- | --- | --- |
| DS1 | Do you have health insurance?  **(1- Yes, 0- No) if no, skip to DS5** | /__/ |
| DS2 | If yes, what is the coverage rate? | /__/__/__/ |
| DS3 | If yes, what is the amount of the monthly contributions (FCFA)? | /__/__/__/__/__/__/__/ |
| DS4 | If so, since when have you been insured? | date /__/__/__/__/ |
| DS5 | In the past month, how much did you spend on:   1. consulting costs? 2. medications (drugs)? 3. hospitalization expenses? 4. Other health expenditures …………………………….. | /__/__/__/__/__/__/__/  /__/__/__/__/__/__/__/  /__/__/__/__/__/__/__/  /__/__/__/__/__/__/__/ |
| DS6 | In the past 12 months, how much did you spend on:   1. consulting costs? 2. medications (drugs)? 3. hospitalization expenses? 4. Other health expenditures …………………………….. | /__/__/__/__/__/__/__/  /__/__/__/__/__/__/__/  /__/__/__/__/__/__/__/  /__/__/__/__/__/__/__/ |
| DS7 | How do you rate your health expenses? **(1-very high; 2- high; 3- moderately high; 4-somewhat high; 5-not at all high)** | /__/ |
