# Supplementary material for: Self-assessed life expectancy among older adults in Côte d’Ivoire
Source: BMC Public Health. 2020 Jun 15;20:932. doi: 10.1186/s12889-020-09034-4 (PMC7296699; doi:10.1186/s12889-020-09034-4)
Supplement: Supplementary file 3 — Additional file 3. This supplementary file is a technical note on the methods used in this paper and cited in the main manuscript – page 9, under the sub-heading, “Estimation of self-assessed life expectancy” of the manuscript. [file 12889_2020_9034_MOESM3_ESM.docx]

# Technical note

We proposed a two-pronged methodological approach consisting of (i) calculating the life expectancy with the SSPs as input [1], and (ii) using a finite mixture of regression models to investigate the determinants of the estimated life expectancy.

The approach used to calculate the life expectancy with the SSPs builds on Bellemare et al. [1] is based on a cubic spline smoothing around each SSP value. This smoothing helps to estimate the cumulative distribution function of the subjective life expectancy for each individual. Given that the cumulative distribution function is strictly monotonic, the function can be approximated by a cubic polynomial around each SSP. Then, the cumulative distribution function of the subjective life expectancy is estimated by connecting these local polynomials. Finally, the average life expectancy for each individual is calculated from the estimated cumulative distribution function. As an example, suppose that a person reports 92 as its SSP for the target age of 70, 83 as its SSP for the target age of 75, 45 as its SSP for the target age of 80 and 10 as its SSP for the target age of 85. Then, on each of the intervals ($\left[ 70;75 \right]$,$\left[ 75;80 \right]$, and $\left[ 80;85 \right]$) the cumulative distribution function is approximated by a cubic polynomial and the junction of these polynomials gives the overall cumulative distribution function for the individual. The technical details for this first stage are presented below:

Let $J_{i}$denotes the number of SSPs available (at least two observed SSPs) are required for individual i. The calculation of the life expectancy is done using the approach by Bellemare et al. [1] and follows these steps:

- Step 1: Use cubic splines to locally approximate the cumulative distribution function around a target age for each individual of the dataset:

$$P\left( Z\leq z_{j} \right)=F\left( z_{j} \right)=a_{j}+b_{j}z_{j}+c_{j}z_{j}^{2}+d_{j}z_{j}^{3}; \forall j=1,\ldots,J_{i} (1)$$

- Step 2: Use the property of continuity of the cumulative function to get the following equations:

$$\left\{ \begin{aligned} {F\left( z_{1} \right)=a_{1}+b_{1}z_{1}+c_{1}z_{1}^{2}+d_{1}z_{1}^{3} (2) \atop F\left( z_{j} \right)=a_{j}+b_{j}z_{j}+c_{j}z_{j}^{2}+d_{j}z_{j}^{3}; \forall j=2,\ldots,J_{i}-1 (3)} \\ {F\left( z_{j} \right)=a_{j+1}+b_{j+1}z_{j}+c_{j+1}z_{j}^{2}+d_{j+1}z_{j}^{3}; \forall j=2,\ldots,J_{i}-1 (4) \atop F\left( z_{J_{i}} \right)=a_{J_{i}-1}+b_{J_{i}-1}z_{J_{i}}+c_{J_{i}-1}z_{J_{i}}^{2}+d_{J_{i}-1}z_{J_{i}}^{3} (5)} \end{aligned} \right.$$

- Step 3: The equation system above is solved to get the parameters ($\forall j=1,\ldots,J_{i}$). Thus, the estimated life expectancy is given by:

$$\hat{E}_{i}\left( Z \right)=\sum_{j=1}^{J_{i}-1} \left[ \left( \frac{\hat{b}_{j}z^{2}}{2}+2*\frac{\hat{c}_{j}z^{3}}{3}+3*\frac{\hat{d}_{j}z^{4}}{4} \right)_{z_{j}}^{z_{j+1}} \right] (6)$$

Once the life expectancy was estimated, we analyzed its determinants by the use of a finite mixture of regression models. This framework allowed modeling the estimated life expectancy generated by a finite number of data generation processes (called herein components/clusters). Let K denote the number of components, y the estimated life expectancy, x a set of relevant explanatory variables, and w a set of variables called concomitant variables that explain the likelihood for an observation to be in each component^2^. Thus, the likelihood function to be estimated is given by:

$$H\left( y | x,\omega,\theta\right)=\sum_{k=1}^{K} \pi_{k}\left( \omega\right)f\left( y|x,\theta_{k} \right) (7)$$

With $\pi_{k}>0 \forall k=1,\ldots,K$ and$\sum_{k=1}^{K} \pi_{k}\left( \omega\right)=1$, and where $\theta_{k}$ denotes the set of parameters for the k^th^ components. The probability $\pi_{k}$denotes the weight of the component k for individual i, i.e. the probability that individual k’s value is distributed per the component k. For each component, the estimated life expectancy is assumed to be normally distributed. The likelihood of the model in Equation 7 is maximized by an Expectation-Maximization (EM) algorithm [2].

The method described above is implemented using the STATA 14 SE and R version 3.4.3 codes below. Stata 14.0 and R version 3.4.3. The significance level was set at 95%.

****************************************

* **BMC Public Health**

* **Richard K. Moussa & Vakaramoko Diaby**

* **richard.moussa@ensea.ed.ci**

****************************************

*******************************************************************

**Step 1:** Stata commands for estimating the subjective life expectancy

(SLE) based on subjective survival probabilities (SSPs). The command

“splinesBBK” has been implemented in Stata by Bissonnette based on

Bellemare et al. (2012) [1].

*******************************************************************

* Read data from file

use " C:/Users/owner/Desktop/ssp_sle/our_data.dta", clear

* NB: The path used above is for illustration purposes. This path shows the location of the data file on the computer, and will differ from a user to another as well as the type of operating system (Windows or Mac). The variables included in the dataset are presented in e-Table 1.

* Use the SSP (vp) at different target age (vk) to estimate the SLE (liftest). The question mark “?” following the variables “vk” and “vp” implies that all variables starting by “vk” and “vp” have to be used.

splinesBBK liftest, qname(qtes) qlist(0.25 0.5 0.75) knots( vk? ) prob( vp? ) eps(0.0001)

####################################################################

**Step 2:** R commands for estimating the determinants of subjective life expectancy (SLE) based on a finite mixture of regression model

####################################################################

# Install packages required for the analysis

install.packages("flexmix"); install.packages("xlsx")

# Load the packages

library("flexmix"); library("xlsx ")

# Read data from file

setwd("C:/Users/owner/Desktop/ssp_sle")

data<- read. xlsx ("our_data.xls")

# NB: The path used above is for illustration purposes. This path shows the location of the data file on the computer, and will differ from a user to another as well as the type of operating system (Windows or Mac). The variables included in the dataset are presented in e-Table 1 (at the end of the codes).

# Estimating the model. Start by setting a seed number to insure that random numbers used in the model will be the same if the model is re-estimated.

set.seed(123)

our_model = flexmix(liftest ~ a05ad + male + maleage1 + union + pobese + rural + toilet1 + seau3 + evacu1 + emploi + etud1 + lrevtete + lrevtete2 + fume + alcool + actphys + nrestrialim, data = data, k = 4, model = FLXglm(family = "gaussian"), concomitant = FLXPmultinom(~ longmal + limact1 + deltas1 + msante + pvie2 + pvie3 + pvie4))

# Providing some statistics about the quality of the model

set.seed(123)

summary(our_model)

# Displaying the estimated parameters for concomitant variables.

set.seed(123)

summary(refit(our_model), which="concomitant")

# Displaying the estimated parameters for each component as well as the estimated variance .

set.seed(123)

summary(refit(our_model))

parameters(our_model, component=1)

parameters(our_model, component=2)

parameters(our_model, component=3)

parameters(our_model, component=4)

# Generating the cluster variable (gclassnew) and the posterior probabilities (gprobnew). The cluster variable indicates the group/cluster in which individuals have been classified.

data$gclassnew <- clusters(our_model)

data$gprobnew <- posterior(our_model)

# Providing statistics on the created clusters/groups. These statistics are related to the posterior probabilities (gprobnew), the estimated life expectancy (liftest), and the age (a05ad).

aggregate(data $gprobnew, list(data$gclassnew), mean)

aggregate(data $liftest, list(data $gclassnew), mean)

aggregate(data $liftest, list(data $gclassnew), sd)

aggregate(data $a05ad, list(data $gclassnew), mean)

aggregate(data $a05ad, list(data $gclassnew), sd)

tabulate(data $gclassnew)

e-Table 1: Variables code names and definition

| **Variable** | **Variable code name** | **Definition** |
| --- | --- | --- |
| **Subject identifier** |  |  |
| Subjective life expectancy | ID_IND | Subject identifier |
| Target age | Vk | Target age for subjective survival probabilities. Four target ages (70, 75, 80, 85) represented by vk1, vk2, vk3, vk4 respectively |
| Survival probabilities | Vp | Subjective survival probabilities for the four target ages (70, 75, 80, 85) represented by vp1, vp2, vp3, vp4 respectively. |
| Age | a05ad | Age in years |
| Male | male | 1 if male, 0 if female |
| Male x age | maleage1 | Interaction variable between male and age |
| Couple | union | 1 if the individual lives with a partner, 0 otherwise |
| Obese | pobese | 1 if obese (Body Mass Index ≥ 30 Kg/m^2^), 0 otherwise |
| Rural | rural | 1 if the individual lives in rural areas, 0 otherwise |
| Low education level | etud1 | 1 if the school level of the individual is at the most primary school level, 0 otherwise |
| Employed | emploi | 1 if the individual is employed, 0 otherwise |
| Income per capita | lrevtete | Household income per capita |
| Income per capita | Lrevtete2 | Second-order term for the variable "Household income per capita" |
| Current smoker | fume | 1 if the individual is currently a smoker, 0 otherwise |
| Alcohol intake | alcool | 1 if the individual consumes at least 2 glasses of alcohol per day, 0 otherwise |
| Exercise/physical activity | Acta Phys | 1 if individual exercises, 0 otherwise |
| Diet | nrestrialim | 1 if the individual observes a diet, 0 otherwise |
| Chronic condition | longmal | 1 if individual reports at least one chronic condition, 0 otherwise |
| Health condition limits working ability | limact1 | 1 if the individual reports that his health condition limits his/her working ability, 0 otherwise |
| Deteriorating health | deltas1 | 1 if the individual report that his health is deteriorating, 0 otherwise |
| Poor health status | msante | 1 if individual report poor health status in general, 0 otherwise |
| Mother alive | pvie2 | 1 if the individual’s mother is alive, 0 otherwise |
| Father alive | pvie3 | 1 if the individual’s father is alive, 0 otherwise |
| Both parents alive | pvie4 | 1 if the individual’s both parents are alive, 0 otherwise |
| Toilet with flush | toilet1 | 1 if the individual has access to a flush toilet, 0 otherwise |
| Underground water | seau3 | 1 if the individual uses underground water as drinking water, 0 otherwise |
| Septic tank | evacu1 | 1 if the individual uses a septic tank for wastes, 0 otherwise |

**References**

1. Bellemare C, Bissonnette L, Kroger S. Flexible approximation of subjective expectations using probability questions. *Journal of Business & Economic Statistics*. 2012;30 (1):125-131.
2. Leisch F. Flexmix: A general framework for finite mixture models and latent class regression in R. Journal of Statistical Software, 11(8):1-17. http://www.jstatsoft.org/v11/i08/.
